# Tyrphostin AG 1024 modulates radiosensitivity in human breast cancer cells

**DOI:** 10.1054/bjoc.2001.2171

**Published:** 2001-12

**Authors:** B Wen, E Deutsch, E Marangoni, V Frascona, L Maggiorella, B Abdulkarim, N Chavaudra, J Bourhis

**Affiliations:** Laboratoire UPRES EA No 27–10 ‘Radiosensibilité-Radiocarcinogénèse Humaine' and Unité METSI, Institut Gustave-Roussy, Villejuif Cédex, 94805, France

**Keywords:** IGF-1R inhibitor, apoptosis, radiosensitivity, breast cancer

## Abstract

Insulin-like growth factor-1 (IGF-1) plays an important growth-promoting effect by activating the PI3K/Akt signalling pathway, inhibiting apoptotic pathways and mediating mitogenic actions. Tyrphostin AG 1024, one selective inhibitor of IGF-1R, was used to evaluate effects on proliferation, radiosensitivity, and radiation-induced cell apoptosis in a human breast cancer cell line MCF-7. Exposure to Tyrphostin AG 1024 inhibited proliferation and induced apoptosis in a time-dependent manner, and the degree of growth inhibition for IC20 plus irradiation (4 Gy) was up to 50% compared to the control. Examination of Tyrphostin AG 1024 effects on radiation response demonstrated a marked enhancement in radiosensitivity and amplification of radiation-induced apoptosis. Western blot analysis indicated that Tyrphostin AG 1024-induced apoptosis was associated with a downregulation of expression of phospho-Akt1, increased expression of Bax, p53 and p21, and a decreased expression of bcl-2 expression, especially when combined with irradiation. To our knowledge, this is the first report showing that an IGF-1 inhibitor was able to markedly increase the response of tumour cells to ionizing radiation. These results suggest that Tyrphostin AG 1024 could be used as a potential therapeutic agent in combination with irradiation.   http://www.bjcancer.com © 2001 Cancer Research Campaign


British Journal of Cancer (2001) 85(12), 2017–2021
© 2001 Cancer Research Campaign
doi: 10.1054/ bjoc.2001.2171, available online at http://www.idealibrary.com on IDEAL

http://www.bjcancer.com

### Tyrphostin AG 1024 modulates radiosensitivity in human breast cancer cells

**B Wen[1,2], E Deutsch[1,2], E Marangoni[1], V Frascona[1], L Maggiorella[1], B Abdulkarim[1], N Chavaudra[1] and J Bourhis**

1Laboratoire UPRES EA No 27–10 'Radiosensibilité-Radiocarcinogénèse Humaine' and Unité METSI, Institut Gustave-Roussy, 94805 Villejuif Cédex France.

**Summary** Insulin-like growth factor-1 (IGF-1) plays an important growth-promoting effect by activating the PI3K/Akt signalling pathway, inhibiting apoptotic pathways and mediating mitogenic actions. Tyrphostin AG 1024, one selective inhibitor of IGF-1R, was used to evaluate effects on proliferation, radiosensitivity, and radiation-induced cell apoptosis in a human breast cancer cell line MCF-7. Exposure to Tyrphostin AG 1024 inhibited proliferation and induced apoptosis in a time-dependent manner, and the degree of growth inhibition for IC20 plus irradiation (4 Gy) was up to 50% compared to the control. Examination of Tyrphostin AG 1024 effects on radiation response demonstrated a marked enhancement in radiosensitivity and amplification of radiation-induced apoptosis. Western blot analysis indicated that Tyrphostin AG 1024-induced apoptosis was associated with a downregulation of expression of phospho-Akt1, increased expression of Bax, p53 and p21, and a decreased expression of bcl-2 expression, especially when combined with irradiation. To our knowledge, this is the first report showing that an IGF-1 inhibitor was able to markedly increase the response of tumour cells to ionizing radiation. These results suggest that Tyrphostin AG 1024 could be used as a potential therapeutic agent in combination with irradiation. © 2001 Cancer Research Campaign http://www.bjcancer.com

**Keywords**: IGF-1R inhibitor; apoptosis; radiosensitivity; breast cancer

### INTRODUCTION

The balance between tumour cell proliferation and tumour cell apoptosis is a critical determinant of malignant tumour outgrowth. To prevent programmed cell death, tumour cells frequently evolve to express secreted growth factors that induce survival signalling pathways, thereby repressing apoptosis. Recent experiments with tumour cell lines and with a transgenic mouse model of β-cell carcinogenesis indicated that insulin-like growth factors (IGFs) act as survival factors to suppress tumour cell apoptosis (Baserga et al, 1997; Rubin and Baserga, 1995; Christofori et al, 1994; Monno et al, 2000).

IGFs are important growth factors in many tumour types. Several lines of evidence indicate that IGF-1 function may be important in the pathogenesis of malignant neoplasms (Sullivan et al, 1995; Kiess et al, 1997; Cianfarani and Rossi, 1997; Zumkeller et al, 1999; Liu et al, 1998). Inhibition of IGF-1 expression in neuroblastoma cells has been shown to induce the regression of established tumours in mice. In addition, overexpression of IGF-1 in neuroblastoma cells appears to prevent apoptosis and enhance neuroblastoma tumorigenesis.

The binding of IGFs to IGF-IR activates the receptor's tyrosine kinase activity, which triggers a cascade of reactions among a number of molecules involved in the signal transduction pathway. One key molecule that is activated in this manner is phosphoinositide 3-kinase (PI3K) (Jones and Clemmons, 1995; LeRoith et al, 1995), a lipid kinase that generates phosphorylated phosphatidylinositol (PI) intermediates (such as PI3, 4,5-triphosphate, PIP3) in the cytosolic leaflet of cellular membranes (Shepherd et al, 1995). These PIP3 intermediates recruit other downstream signalling molecules, particularly serine/threonine protein kinases such as AKT and atypical protein kinase C (PKC) isoforms to membranes. Membrane recruitment of these molecules facilitates their interaction with upstream regulators, including the serine kinase 3-phosphoinositide-dependent kinase 1 (PDK1), which phosphorylate and activate them.

Other signal transduction pathways that are initiated by IGF-1R may also exist (Lopaczynski et al, 1999). Activation of IGF-1R by ligand binding is required for IGF-1R to mediate the actions of IGFs. In addition to mediating the mitogenic and anti-apoptotic actions of IGFs, IGF-1 is involved in cell transformation. In vitro experiments have shown that removal of IGF-1R from the cell membrane by eliminating the IGF-1R gene, by suppressing its expression or by inhibiting its function, can abolish cell transformation (Baserga et al, 1995).

Some tumour-suppressor gene products have a profound impact on the IGF family. Wild-type p53 protein induces the expression of IGF binding protein-3 (IGFBP-3), represses the transcription of IGF-II and suppresses IGF-1 expression (Buckbinder et al, 1995; Zhang et al, 1996, 1998; Werner et al, 1996; Webster et al, 1996; Ohlsson et al, 1998). When IGF-1-induced DNA synthesis takes place in breast cancer cells, p53 can lose its function by undergoing phosphorylation and relocation from the nucleus to the cytoplasm (Takahashi et al, 1993).

In the past decade, a family of low-molecular-weight compounds, Tyrphostins, have been synthesized and identified as potent inhibitors of protein tyrosine kinases (PTKs), and different members of the Tyrphostin family recognize PTKs of different growth factor receptors such as epidermal growth factor receptor

Received 11 May 2001
Revised 25 September 2001
Accepted 28 September 2001

Correspondence to: J Bourhis

[2]These authors contributed equally to the work.

(EGFR), insulin-like growth factor receptor (IGF-1R) in a selective manner (Gazit et al, 1989).

Tyrphostin AG 1024, one tyrosine kinase inhibitor specifically targeting IGF-1 receptor, is metabolized intracellularly, creating substances with increased activity towards the receptor and down-regulated activity of Akt kinase. The objective of this study was to examine the potential therapeutic purpose of Tyrphostin AG 1024 in combination with irradiation to counteract tumour cell proliferation and to modulate cellular radiosensitivity and apoptosis in a human breast cancer cell line, MCF-7.

### MATERIALS AND METHODS

### Materials

All materials and chemicals were purchased from Sigma (St Louis, MC, USA) unless noted otherwise. The inhibitor of IGF-1R, Tyrphostin AG 1024 was purchased from Alexis Biochemicals. Akt, phospho-Akt, and NIH-3T3 PDGF-treated protein were purchased from New England Biolabs, Inc. Bax and bcl-2, and p21 antibodies were purchased from Santa Cruz Biotechnology, Inc.

Horseradish peroxidase-linked anti-rabbit and anti-mouse antibody were from Jackson ImmunoResearch Laboratory Inc.; molecular markers and fetal bovine serum were from Biological Industries and ECL was purchased from Amersham Biotech Com. ApopTag™ Plus apoptosis Detection Kit-Fluorescence was purchased from Oncor Inc.

### Cell line

MCH-7 was maintained in RPMI 1640 medium supplemented with 10% fetal bovine serum, 100 U/ml of penicillin and streptomycin, 2 mM L-glutamine at 37˚C in a humidified atmosphere of 5% $CO_2$.

### IGF1R inhibitor treatment

The anti-proliferative effect of Tyrphostin AG 1024 in vitro was examined following replicate plating of a known number of single cells. After incubation of 24 h, 10 nM/ml Tyrphostin AG 1024 were added to the medium and cells were trypsinized, stained with trypan blue and counted with a haemocytometer at indicated time intervals.

### Clonogenic survival

Survival following exposure to irradiation and inhibitor of IGF-1R Tyrphostin AG 1024 was defined as the ability of the cells to maintain their clonogenic capacity and form colonies. Briefly, after treating with Tyrphostin AG 1024 10 nM/ml for 48 h, cells were trypsinized, counted, seeded, and irradiated for colony formation, or irradiated first; cells were trypsinized, counted and seeded, and then exposed to Tyrphostin AG 1024 for 48 h for colony formation. Following an incubation interval of 2 weeks, colonies were stained with crystal violet and manually counted. Colonies consisting of ≥ 50 cells were scored. Differences in the percentage of cell survivals among treatment groups were assessed by two-sided student test.

### Apoptosis analysis (TUNEL assay)

Apoptosis was evaluated by dual staining of MCF-7 with fluoresceine anti-digoxigenin and propidium iodide, as previously described (Schmitz, 1991). Briefly, fixed cells were washed with PBS, suspended in TdT buffer with TdT enzyme and Dig-dUTP for 60 min, and suspended in FITC blocking solution with anti-Dig-Fluorescein for 30 min at room temperature and kept in a dark place. Cells were then rinsed in buffer and resuspended in propidium iodide/RNase A solution for 30 min then analyzed by flow cytometry.

### *Expression of phospho-Akt1, Bax, p53, bcl-2 and p21*

#### *Western blot*

After treatment, cells were lysed with Tween-20 lysis buffer (50 mM HEPES pH 7.4, 15 mM NaCl, 0.1% Tween-20, 10% glycerol, 2.5 mM EGTA, 1 mM EDTA, 1 mM phenylmethysulfonyl fluoride, and inhibitor for proteinases) and sonicated. Equal amounts of proteins were analyzed by SDS-PAGE. Thereafter, proteins were transferred to nitrocellulose membranes and analyzed by specific antibodies against phospho-Akt1 (ser473), Bax and β-actin. Proteins were detected via incubation with horseradish peroxidase-conjugated secondary antibodies and the enhanced chemiluminescence detection system. β-actin was used as a control for loading.

### RESULTS

### Proliferation inhibited by Tyrphostin AG 1024

Proliferation curves were evaluated in MCF-7 following the addition of an inhibitor of IGF-lR Tyrphostin AG 1024 with a different time interval and irradiation. The growth inhibition profiles over the 5-day exposure period are shown in Figure 1. Exposure to Tyrphostin AG 1024 and irradiation inhibited proliferation of MCF-7 in a time-dependent manner. This effect was more pronounced when Tyrphostin AG 1024 was combined with irradiation. In both the pre-irradiation and post-irradiation exposure settings, treatment with Tyrphostin AG 1024 induced a marked decrease of proliferation.

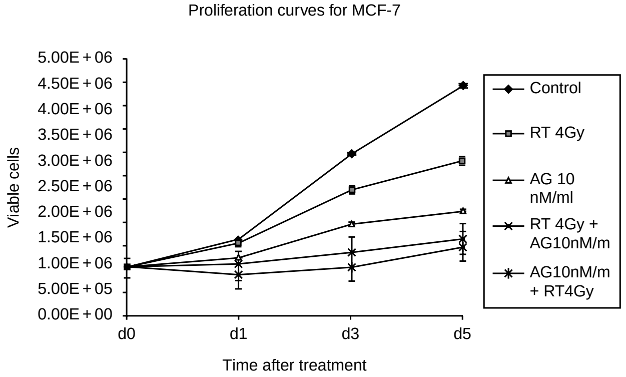

**Figure 1** Tyrphostin AG 1024 inhibited proliferation of MCF-7, especially when combined with irradiation. Known numbers of single cells from MCF-7 were seeded into 35-mmΦ dishes. After 24 hours, incubation, cells were treated with AG 1024 10 nM/ml, irradiated with 4 Gy, AG 1024 10 nM/ml combined with irradiation or irradiation combined with AG 1024 with 6 hours' interval. Cells were harvested at different intervals and counted with trypan blue dye exclusion

 © 2001 Cancer Research Campaign

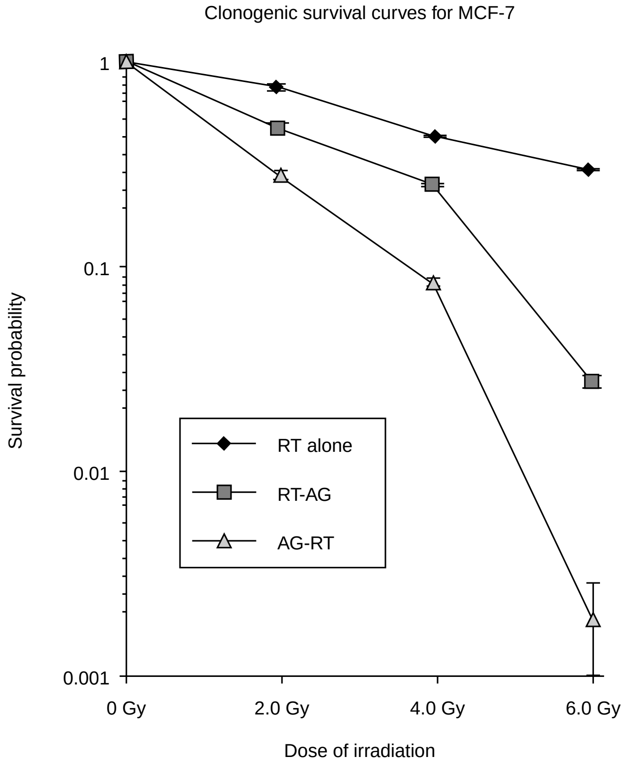

**Figure 2** Tyrphostin AG 1024 enhanced radiosensitivity. The influence of Tyrphostin AG 1024 on intrinsic radiosensitivity was examined by clonogenic survival in the MCF-7 cell line. MCF-7 cells were exposed to Tyrphostin AG 1024 10 nM/ml for 2 d before or after irradiation. Control cells were exposed to irradiation without Tyrphostin AG 1024

### Radiosensitivity enhanced by Tyrphostin AG 1024

Experiments were designed to determine the effects of Tyrphostin AG 1024 response combined with irradiation on clonogenic survival of MCF-7 cells. Figure 2 depicts radiation-related survival curves for cells exposed to Tyrphostin AG 1024 before or after exposure to irradiation. Exposure of MCF-7 to 10 nM/ml Tyrphostin AG 1024 alone induced a reduction in plating efficiency of about 20%. Increased cell killing was obtained using Tyrphostin AG 1024 combined with pre- or post-irradiation. The efficacy on cell killing was more pronounced when the inhibitor was followed by irradiation as compared to irradiation followed by the inhibitor ($P < 0.05$).

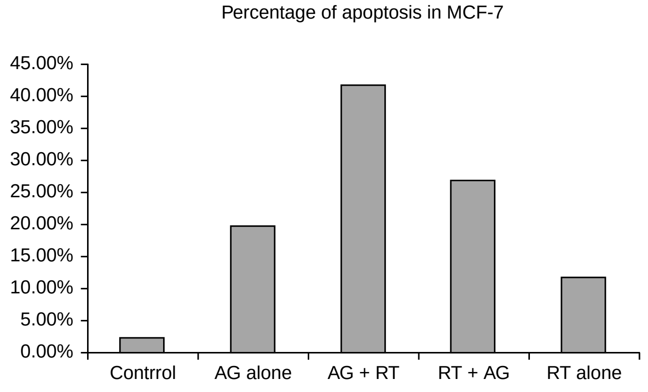

**Figure 3** Apoptosis was induced by Tyrphostin AG 1024 10 nM/ml and/or irradiation 10 Gy at 48 h

### Apoptosis induced by Tyrphostin AG 1024

To determine whether apoptotic response was influenced by Tyrphostin AG 1024 and/or irradiation, MCF-7 cells were exposed to Tyrphostin AG 1024 10 nM/ml, irradiation (10 Gy) or both. As shown in Figure 3, the percentage of apoptotic cells was 20.1%, 11.8% when cells were exposed to Tyrphostin AG 1024 alone or irradiation alone, respectively, and was markedly increased when Tyrphostin AG 1024 was combined with irradiation, especially when cells were exposed first to Tyrphostin AG 1024 and irradiated thereafter.

### Expression of phospho-Akt1 (ser473) influenced by Tyrphostin AG 1024 in a time-responsive manner

To measure the amount of phospho-Akt1, MCF-7 cells were exposed to Tyrphostin AG 1024, lysed and immunoblotted for phosphotyrosine-containing protein of 60 kD. As shown in Figure 4A, phospho-Akt1 was downregulated by Tyrphostin AG 1024 in a time-responsive manner but the amount of Akt remained the same (data not shown). The expression of phospho-Akt1 was more downregulated when cells were treated with starvation 24 h before adding the inhibitor (data not shown). The expression of phospho-Akt1 was not influenced by irradiation alone (Figure 4A). Figure 4B shows that Tyrphostin AG 1024 downregulated phospho-Akt1, and this effect was more pronounced when combined with irradiation.

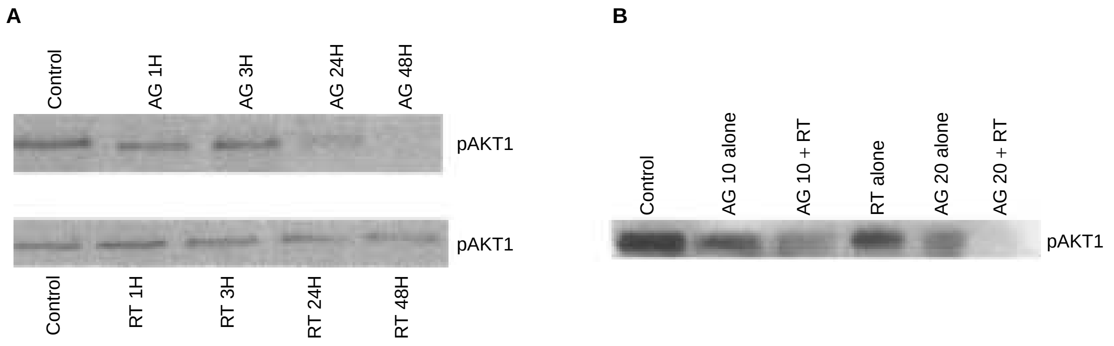

**Figure 4** Expression and modulation of phospho-Akt1. (**A**) MCF-7 cells were treated with Tyrphostin AG 1024 10 nM/ml or irradiated with 4 Gy at defined time interval. (**B**) MCF-7 cells were treated with 10 nM/ml, 20 nM/ml of Tyrphostin AG 1024, and/or combined with irradiation with 10 Gy at a 24-hour intervals

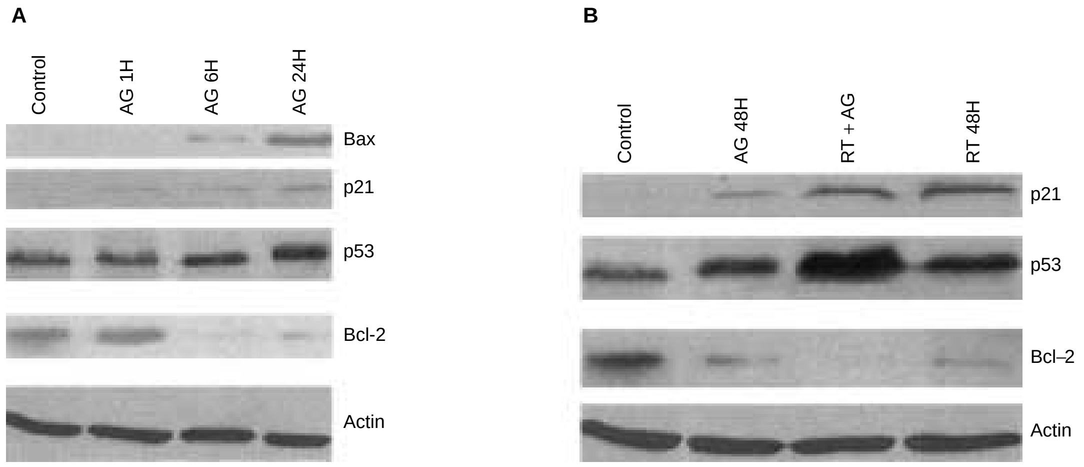

**Figure 5** Effect of Tyrphostin AG 1024 alone or with irradiation on the expression of Bax, p53, bcl-2 and p21. (**A**) MCF-7 cells were treated with 10 nM/ml Tyrphostin AG 1024 at defined time interval. (**B**) MCF-7 cells were treated with 10 nM/ml of Tyrphostin AG 1024, combined with irradiation 10 Gy at a 24-hour intervals, and then harvested and lysed at 48 h

### Increased expression of Bax, p53 and p21, and decreased expression of bcl-2 after Tyrphostin AG 1024 exposure

To further investigate the effect of Tyrphostin AG 1024 on MCF-7, we performed Western blots to examine the expression of Bax, bcl-2, p21 and p53, known as critical regulators of apoptosis and the cell cycle. As shown in Figure 5, treatment of MCF-7 cells with Tyrphostin AG 1024 increased Bax, p21 and p53 expression, and decreased the expression of bcl-2, especially when combined with irradiation.

### DISCUSSION

The proliferation of tumour cells during irradiation has been identified as a factor that adversely impacts tumour response and local control (Fowler and Lindstrom, 1992). The approaches to reduce the impact of tumour cell repopulation during radiation therapy involve the delivery of biological agents that could reduce or inhibit tumour cell proliferation. Modalities of treatment based on growth factor-receptor interactions or on interference with their signal transduction pathways, could be promising therapeutic possibilities (Huang et al, 1999). Here, we investigated the ability of Tyrphostin AG 1024, one inhibitor of IGF-1R, to inhibit proliferation and also examined the capacity of Tyrphostin AG 1024 to modulate radiosensitivity in a breast carcinoma cell line, MCF-7.

The results have shown that Tyrphostin AG 1024 markedly inhibited growth of MCF-7 cells (Figure 1). This growth inhibition was associated with the induction of apoptosis. We also observed that exposure of MCF-7 cells to the inhibitor of IGF-1 induced a decreased expression of phospho-Akt1. These results were in good agreement with those of Dupont et al, showing that IGF-1 acted to increase P13K activity and Akt phosphorylation (Dupont et al, 2000). Yuan had also showed that apoptosis was induced in cancer cells by inhibiting the PI3-kinase Akt pathway (Yuan et al, 2000).

Our results are in agreement with previous studies, suggesting that inactivation of IGF-1 function by the inhibitor could be essential for induction of apoptosis. Firstly, overexpression of Akt inhibits apoptosis induced by various stimuli (Yuan et al, 2000). Secondly, treatment of tumour with antibody or anti-sense oligonucleotides targeting IGF-1R could decrease Akt protein expression and sensitize cells to drug- or irradiated-induced programmed cell death (Trojan et al, 1994; Shevelev et al, 1997; and Scotlandi et al, 1998). Finally, our results from kinetics experiments showed that the levels and tyrosine activity of Akt1 were decreased before MCF-7 apoptotic cell death induced the by IGF-1 inhibitor.

Many components of the biological response to ionizing radiation in mammalian cells are mediated through signal transduction, cell cycle regulation, and DNA repair pathways (Coleman et al, 1996). Growth factors may play an important role in modulating these cellular responses to irradiation. Indeed some reports have shown a marked effect of epidermal growth factor (EGF) on the radiosensitivity of cancer cells. For example, Wollman reported that pre-irradiation exposure to EGF enhanced radioresistance in MCF-7 cells, whereas Huang et al (1999) showed that exposure to C225, an anti-EGF receptor monoclonal antibody, either before or following irradiation, enhanced the radiosensitivity in a human squamous cell carcinoma.

So far, there was no report evaluating the effect of IGF-1R inhibitor on the radiosensitivity of cancer cells. Here, we report for the first time that exposure to inhibitor IGF-1R, Tyrphostin AG 1024, could enhance radiosensitivity in MCF-7 cells. It was possible that the enhancement of radiosensitivity by Tyrphostin AG 1024 could be mediated by mechanisms involving inhibition of Akt phosphorylation and induction of apoptosis by increasing expression of Bax and p53, and decreasing expression of bcl-2 (Figures 4 and 5). Indeed our results are in agreement with those of previous studies showing that IGF-1 could inhibit apoptosis, stimulate the expression of bcl-2 protein and in parallel suppress Bax expression, which resulted in an increase in the relative amount of the bcl/Bax heterodimer, thereby blocking initiation of the apoptotic pathway (Minshall et al, 1997; Parrizas et al, 1997; Wang et al, 1998).

Here, for the first time we showed that Tyrphostin AG 1024 can increase p21 expression, which can lead to a negative regulation of cell proliferation (Figure 5). Indeed in a number of studies that had used various cell lines, p21 has been described as a negative regulator of cell cycle and proliferation. However these results showing an increase of p21 after IGF-1R inhibitor exposure are not in agreement with the data reported by Dupont et al (2000) since they showed that IGF-1 was able to increase moderately the level of p21 by interfering directly with the p21 promoter.

© 2001 Cancer Research Campaign

A possible explanation for this discrepancy might be that p53 expression was induced by AG 1024, which consequently would increase the level of p21 (El-Deiry et al, 1993; Kim, 1997; Maestro et al, 1997).

In conclusion, our findings indicated that Tyrphostin AG 1024, IGF-IR inhibitor, was an effective anti-proliferative agent for breast carcinoma cells MCF-7. Growth inhibition was associated with induction of apoptosis and an inhibition AKT/PI3 activity, increased expression of Bax, p53 and p21 and decreased expression of bcl-2. In addition, this is the first report to show that an IGF-1 inhibitor was able to markedly increase the response of tumour cells to ionizing radiation.

### ACKNOWLEDGEMENTS

We thank Arlette Vervish for her assistance in the flow cytometry facility at Paul Brousse Hospital, CNRS, France. This work was supported by a grant from the Institut Fédératif de Recherche IFR No. 54.

### REFERENCES

Baserga R (1995) The insulin-like growth factor I receptor: a key to tumor growth? *Cancer Res* **55**: 249–252

Baserga R, Hongo A, Rubini M, Prisco M and Valentinis B (1997) The IGF-1 receptor in cell growth, transformation and apoptosis. *Biochim Acta* **1332**: 105–126

Blume-Jensen P and Hunter T (2001) Oncogenic kinase signaling. *Nature* **411**: 355–365

Buckbinder L, Talbott R, Velasco-Miguel S, Takenaka I, Faha B and Seizinger BR (1995) Induction of the growth inhibitor IGF-binding protein 3 by p53. *Nature* **377**: 646–649

Cianfarani S and Rossi P (1997) Neuroblastoma and insulin-like growth factor system. *Eur J Pediatr* **156**: 256–261

Coleman CN and Stevenson MA (1996) Biologic basis for radiation oncology. *Oncology* **10**: 399–411

Christofori G, Naik P and Hanahan D (1994) A second signal supplied by insulin-like growth factor II in oncogene-induced tumorigenesis. *Nature* **369**: 414–418

Dupont J, Karas M and LeRoiths D (2000) The potentiation of estrogen on insulin-like growth factor I action in MCF7 human breast cancer cells includes cell cycle components. *J Biol Chem* **275**: 35893–35901

El-Deiry WS, Tokino T, Velculescu VE, Levy DB, Parsons R, Trent JM, Lin D, Mercer WE, Kinzler KW and Vogelstein B (1993) Wafl, a potential mediator of p53 tumor suppression. *Cell* **75**: 817–825

Fowler JF and Lindstrom MJ (1992) Loss of local control with prolongation in radiotherapy. *Int J Radiat Oncol Biol Phys* **23**: 457–467

Gazit A, Yaish P and Gilon C (1989) Tyrphostins I. Synthesis and biological activity of protein tyrosine linase inhibitors. *J Med Chem* **32**: 2344–2352

Huang SM, Bock JM and Harari PM (1999) Epidermal growth factor receptor blockade with C225 modulates proliferation, apoptosis, and radiosensitivity in squamous cell carcinomas of the head and neck. *Cancer Res* **59**: 1935–1940

Jones JI and Clemmons DR (1995) Insulin-like growth factors and their binding proteins: biological actions. *Endocr Rev* **16**: 3–34

Kiess W, Koepf G, Christiansen H and Blum WF (1997) Human neuroblastoma cells use either insulin-like growth factor-I or insulin-like growth factor-II in an autocrine pathway via the IGF-1 receptor: variability of IGF, IGF binding protein (IGFBP) and IGF receptor gene expression and IGF and IGFBP secretion in human neuroblastoma cells in relation to cellular proliferation. *Regul Pept* **72**: 19–29

Kim TK (1997) In vitro transcriptional activation of p21 promoter by p53. *Biochem Biophys Res Commun* **234**: 300–302

Lee AV, Gooch JL, Oesterreich S, Guler RL and Yee D (2000) Insulin-like growth factor I-induced degradation of insulin receptor substrate 1 is mediated by the 26S proteasome and blocked by phosphatidylinositol 3′-kinase inhibition. *Mol Cell Biol* **20**: 1489–1496

Liu X, Turbyville T, Fritz A and Whitesell L (1998) Inhibition of insulin-like growth factor expression in neuroblastoma cells induces the regression of established tumors in mice. *Cancer Res* **58**: 5432–5438

LeRoith D, Werner H, Beitner-Johnson D and Roberts CT Jr. (1995) Molecular and cellular aspects of the insulin-like growth factor 1 receptor. *Endocr Rev* **16**: 143–163

Levitzki A and Gazit A (1995) Tyropsine kinase inhibition: an approach to drug development. *Science* **267**: 1782–1788

Lopacznski W (1999) Different regulation of signaling pathways for insulin and insulin like growth factor I. *Acta Biochim Pol* **46**: 51–60

Maestro R, Gloghini A, Doglioni C, Piccinin S, Vukosavljevic T, Gasparotto D, Carbone A and Biocchi M (1997) Human non-Hodgkin's lymphomas overexpress a wild-type form of p53 which is a functional transcriptional activator of the cyclin-dependent kinase inhibitor p21. *Blood* **89**: 2523–2528

Minshall C, Arkins S, Straza J, Conners J, Dantzer R, Freund GG et al (1997) IL-4 and insulin-like growth factor-I inhibit the decline in Bcl-2 and promote the survival of IL-3-deprived myeloid progenitors. *J Immunol* **159**: 1225–1232

Monno S, Newman MV, Cook M and Lowe WL Jr. (2000) Insulin-like growth factor I activates c-Jun N-terminal kinase in MCF7 breast cancer cells. *Endocrinology* **141 (2)**: 544–550

Ohlsson C, Kley N, Werner H and LeRoith D (1998) p53 regulates insulin-like growth factor-I (IGF-I) receptor expression and IGF-I-induced tyrosine phosphorylation in an osteosarcoma cell line: interaction between p53 and Sp1. *Endocrinology* **139**: 1101–1107

Parrizas M and LeRoith D (1997) Insulin-like growth factor-1 inhibition of apoptosis is associated with increased expression of the bcl-xL gene product. *Endocrinology* **138**: 1355–1358

Rubin R and Baserga R (1995) Biology of desease: insulin-like growth factor-1 receptor. *Lab Invest* **73**: 311–331

Schmitz G (1991) Nonradioactive labelling of oligonucleotides in vitro with the hapten digoxigenin by tailing with terminal transferase. *Anal Biochem* **192**: 222–231

Sphepherd PR, Withers DJ and Siddle K (1998) Phosphoinositide 3-kinase: the key switch mechanism in insulin signaling. *Biochem J* **333**: 471–470

Scotlandi K, Benini S, Nanni P, Lolliai P, Nicoletti G, Landuzzi L, Serra M, Manara C, Picci P and Baldini N (1998) Blockage of insulin-like growth factor-I receptor inhibits the growth of Ewing's sarcoma in athymic mice. *Cancer Res* **58**: 4127–4131

Shevelev A, Burfeind P, Schulze E, Rininsland F, Johnson T, Trojan J, Chernicky C, Hélène C, Ilan J and Ilan J (1997) Potential triple helix-mediated inhibition of IGF-I gene expression significantly reduces tumorigenicity of glioblastoma in an animal model. *Cancer Gene Ther* **4**: 105–112

Sullivan KA, Castle VP, Hanash SM and Feldman EL (1995) Insulin-like growth factor II in the pathogenesis of human neuroblastoma. *Am J Pathol* **147**: 1790–1798

Takahashi K and Suzuki K (1993) Association of insulin-like growth-factor-I-induced DNA synthesis with phosphorylation and nuclear exclusion of p53 in human breast cancer MCF-7 cells. *Int J Cancer* **55**: 453–458

Trojan J, Johnson T, Rudin S, Blossey B, Kelley K, Shevelev A, Abdul-Karim F, Anthony D, Tykocinski M, Ilan J and Ilan J (1994) Gene therapy of murine teratocarcinoma: sepatate functions for insulin-like growth factors I and II in immunogenicity and differentiation. *Proc Natl Acad Sci USA* **91**: 6088–6092

Wang L, Ma W, Markovich R, Lee WL and Wang PH (1998) Insulin-like growth factor I modulates induction of apoptotic signaling in H9C2 cardiac muscle cells. *Endocrinology* **139**: 1354–1360

Werner H, Karnieli E, Rauscher FJ and LeRoith D (1996) Wild-type and mutant p53 differentially regulate transcription of the insulin-like growth factor I receptor gene. *Proc Natl Acad Sci USA* **93**: 8318–8323

Webster NJ, Resnik JL, Reichart DB, Strauss B, Haas M and Seely BL (1996) Repression of the insulin receptor promoter by the tumor suppressor gene product p53: a possible mechanism for receptor overexpression in breast cancer. *Cancer Res* **56**: 2781–2788

Xiong Y, Hannon GJ, Zhang H, Casso D, Kobayashi R and Beach D (1993) p21 is a universal inhibitor of cyclin kinases. *Nature* **366(6456)**: 701–704

Yuan Z, Sun M,Feldman R, Wang G, Ma X, Jiang C, Coppola D, Nicosia A and Cheng J (2000) Frequent activation of $AKT^2$ and induction of apoptosis by inhibition of phosphoinositide-3-OH kinase/Akt pathway in human ovarian cancer. *Oncogene* **19**: 2324–2330

Zhang L, Kashanchi F, Zhan Q, Zhan S, Brady JN, Fornace AJ et al (1996) Regulation of insulin-like growth factor II P3 promoter by p53: a potential mechanism for tumorigenesis. *Cancer Res* **56**: 1367–1373

Zhang L, Zhan Q, Zhan S, Kashanchi F, Fornace AJ Jr., Seth P et al (1998) p53 regulates human insulin-like growth factor II gene expression through active P4 promoter in rhabdomyosarcoma cells. *DNA Cell Biol* **17**: 125–131

Zumkeller W and Schwab M (1999) Insulin-like growth factor system in neuroblastoma tumorigenesis and apoptosis: potential diagnostic and therapeutic perspectives. *Horm Metab Res* **31**: 138–141

*© 2001 Cancer Research Campaign*
